# 3D Printing with Marine Gelatin: A Cross-Sector Review of Biomedical, Food, and Health Uses

**DOI:** 10.3390/md24060217

**Published:** 2026-06-16

**Authors:** Beril Bayrak, Andrew Cashman, Patrick McGowan, Julie Maguire, Saravana Periaswamy Sivagnanam

**Affiliations:** 1 Department of Biological Sciences, Munster Technological University, T12 P928 Cork, Ireland; beril.bayrak@mymtu.ie; 2 Department of Mechanical, Biomedical and Manufacturing Engineering, Munster Technological University, T12 P928 Cork, Ireland; andrew.cashman@mtu.ie (A.C.); paddy.mcgowan@mtu.ie (P.M.); 3 Bantry Marine Research Station, P75 AX07 Cork, Ireland; jmaguire@bmrs.ie

**Keywords:** marine gelatin, 3D printing, bioinks, rheology, extrusion-based bioprinting, tissue engineering

## Abstract

Gelatin is a valuable hydrocolloid produced by partial hydrolysis of collagen from mainly mammalian and fish sources. The rheological properties of fish gelatin differ from those of mammalian species in terms of gel strength, viscosity, and other rheological characteristics, even from different fish species and parts of the fish with different properties. Fish gelatin is sustainable for the environment and easy for people to accept for cultural reasons. Owing to these properties, gelatin is used across food, biomedical, pharmaceutical, and health sectors, where 3D printing enables customization and functional performance. Key determinants of print fidelity include gelatin concentration, rheological properties, temperature, gelling behavior, water content, and printing parameters. Suitability for 3D printing is typically assessed via physicochemical characterization, particularly rheology and gelling mechanisms/kinetics. Gelatin-based 3D printing systems offer various advantages due to their biocompatibility, low cost, and controllable rheological properties, and they have potential applications in the food, healthcare, biomedical, tissue engineering, and drug delivery system areas. Using gelatin in combination with other additives can improve printing accuracy and mechanical strength parameters, overcome the limitations of gelatin’s inherent mechanical strength, and develop higher printing accuracy and performance systems. This allows for the development of functional, innovative, and high-value-added products while ensuring safe use.

## 1. Introduction

**Figure 1:**
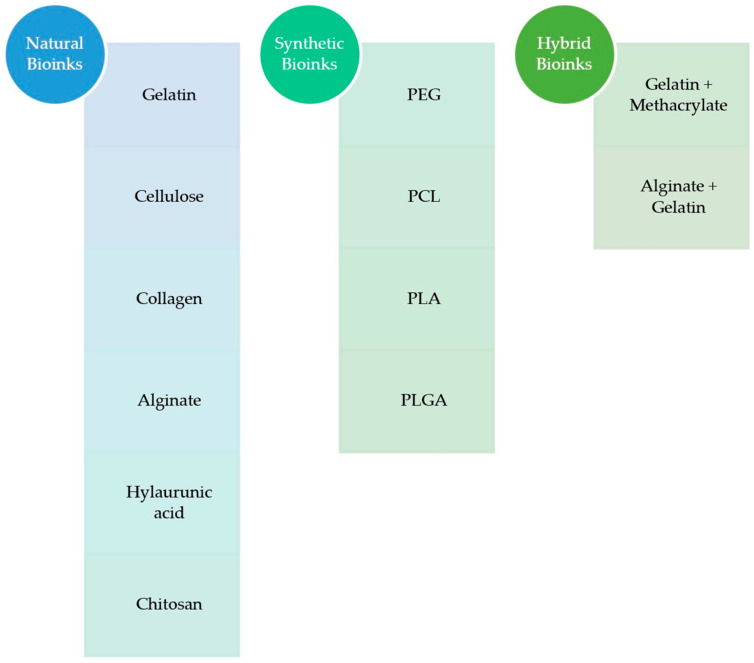
Classification and properties of bioinks: natural, synthetic, and hybrid types [9,10,11].

Gelatin can be obtained from different sources, such as mammalian (bovine and porcine), insects, and fish [1]. Fish gelatin, produced by acid extraction since 1960, is widely used today. It is obtained particularly from the skin, scales, and bones of cold-water fish such as cod, haddock, and salmon, and from warm-water fish such as tuna, tilapia, and catfish [2]. Fish gelatin, besides being a promising alternative to mammalian gelatin, can add economic value to fish by-products and reduce waste from the seafood industry. However, nowadays, mammalian gelatins are still used more than fish gelatin, and one of the reasons for this may be the presence of a fishy odor. In some studies, this issue has been improved in gelatin extraction by adding an additional filtration step or pre-treatments with salt, base, or acid. Another reason is the perception that fish gelatin’s gelling ability performs lower than mammalian gelatin, but this situation varies depending on the type of fish chosen and environmental factors [3]. The main objectives in promoting these processes are (1) to evaluate the recovery and sustainability potential of food by-products and (2) waste generated during harvesting and post-harvest processing; to (3) examine the development of 3D printing inks through the recovery of by-products and waste; (4) to assess the performance of 3D printing technology, its impact on customized and personalized sustainable food production, and the functionality of these resources as biodegradable materials in the packaging industry [4]. Especially in recent years, 3D printing has been cited as a very powerful production tool in the biomedical, food, and healthcare fields. Production occurs through layer-by-layer deposition, aiming for the efficient use of proteins and living cells. This type of production allows for the creation of personalized products at a low cost. One of its most important advantages is the ability to produce devices and nutrition tailored to the needs of individual patients, thus providing them with a more comfortable life. Three-dimensional printing allows for rapid prototyping, enabling quick production, testing, and market launch. Unlike traditional manufacturing, 3D printing allows for the production of complex structures that are difficult to manufacture, thereby improving the functionality of implants and tissue scaffolds. These types of new solutions improve healthcare services and allow for positive patient feedback. In biomedical applications, rapid prototyping, personalization options, and improved therapeutic outcomes are excellent opportunities for incorporating 3D printing into the biomedical industry. This technology allows for the creation of implants, prostheses, and surgical instruments tailored to an individual’s anatomy and with enhanced functionality. Another area of application is dental implant procedures, where it involves time-consuming and costly traditional methods such as casting, milling, and polishing, but precise and sharp designs can be achieved with 3D printing [5]. Various biopolymers like gelatin, collagen, alginate, hyaluronic acid, chitosan, PEG, PLA, and combinations of these successfully compete in the global market thanks to their unique characteristics, which generate significant demand in biomedical applications in 3D printing (Figure 1). Furthermore, it is no surprise that improving human health and lifespan contributes to one of the fastest-growing markets for tissue engineering and regenerative medicine products. To help this end, the industry is developing new biomaterial-based products, including both synthetic and naturally sourced materials [6]. Gelatin is one of the functional biopolymers widely used, particularly for regulating elasticity, consistency, and stability, and can be obtained from the skin and bones of land animals, as well as fish and insects [1]. In recent years, gelatins derived from fish and edible insects have provided an acceptable alternative source for halal and kosher products and offer an alternative solution for markets concerned about bovine spongiform encephalopathy (BSE). It has been reported that insect-derived gelatin can be used in ice cream production [1,7]. Also, BSE is particularly used in pharmaceutical, biomedical, and food fields due to its low cost, wide availability, biocompatibility, degradability, gelling capacity, and food compatibility [8].

Each of the hydrocolloid components affect system performance in different ways. The benefits and limitations of these components vary depending on the application area.

**Figure 2:**
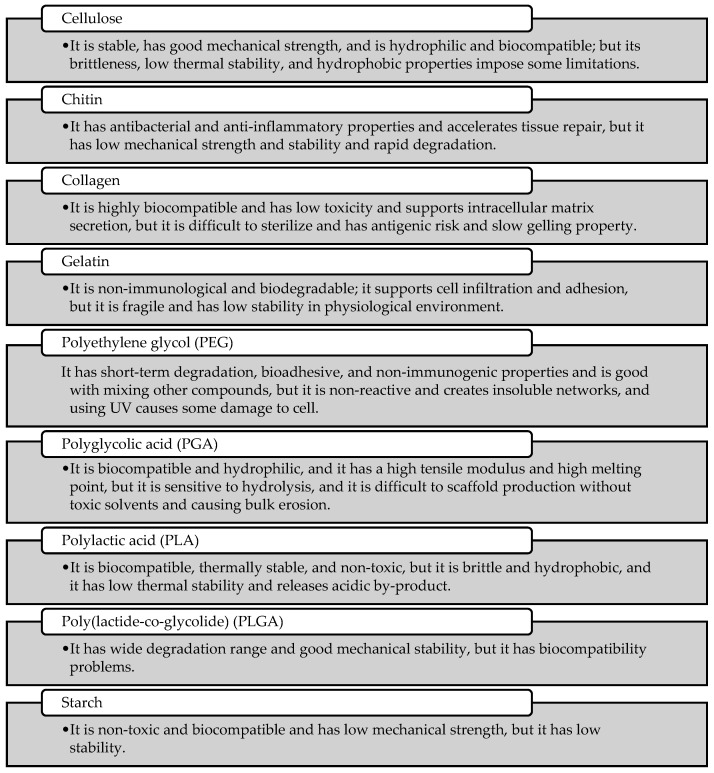
Hydrogel properties for bioinks [6,14].

In this context, the advantages and disadvantages of these components are shown in Figure 2. The world’s most common natural organic polymer, cellulose, is a simple polymer that is not digested by the human body, but it forms insoluble crystalline microfibrils that are quite resistant to enzymatic hydrolysis and renowned for its eco-friendliness, biodegradability, renewability, and biocompatibility. Due to its many reactive groups, cellulose is a biomaterial that is easy to cross-link and modify [10,12,13]. Large brown algae are the primary source of alginic acid used in alginate production. Alginate has low production costs and good gel properties. It is typically found in the form of cationic salts, including magnesium (Mg^2+^), barium (Ba^2+^), sodium (Na^+^), and others. Sodium alginate is the most commonly used alginate agent [10]. The use of pectin in the pharmaceutical and biotechnology sectors is also increasing. This naturally occurring polysaccharide is essential for the structural integrity of plant cell walls. Unlike other polysaccharides, pectin is safe to consume and spontaneously degrades [10]. Hyaluronic acid (HA) is a naturally occurring polysaccharide with diverse sources and strong biocompatibility. A key component of the human body, HA, unlike other polysaccharides, is obtained from animal tissues such as chicken crowns and bovine vitreous bodies. Starch is a neutral polysaccharide produced by plants such as rice, wheat, and corn; it is in the form of insoluble granules, which are its main energy source [10]. The temperature-dependent conformations of xanthan gum in aqueous solutions are as follows: through gel-like activity, it exhibits a stable and ordered double helix chain structure at low temperatures (below 40–50 °C); at higher temperatures (above 50 °C), it exhibits a flexible and disordered helical shape [10]. Environmental impact, recyclability, biodegradability, toxicity, and sustainability of polymers in 3D biological manufacturing: While a wide variety of polymers are used in 3D biological manufacturing due to their mechanical versatility and biocompatibility, it is equally important to consider their broader environmental impacts. Synthetic polymers such as polyethylene glycol (PEG) are not inherently biodegradable, although large-scale disposal still presents environmental challenges. Natural polymers, including alginate, chitosan, gelatin, silk fibroin, and hyaluronic acid, generally have strong sustainability profiles as they decompose into renewable, biodegradable, and non-toxic products [9].

The advantages of plant-based polymers such as alginate, pectin, starch, and cellulose derivatives include low cost, wide availability, and the absence of ethical or religious concerns. Alginate and pectin hydrogels possess favorable biocompatibility properties and are used in food and biomedical products. Furthermore, unlike gelatin, plant-derived polymers generally lack natural cell adhesion motifs, limiting their ability to mimic the natural extracellular matrix without chemical modification [15]. In the food industry, polysaccharides such as gellan, alginate, or carrageenan-based gels are used as alternatives to gelatin, and they generally have less flexible molecular backbones, thus withstanding higher hot viscosities than gelatin. Methoxylpectin is used as a gelatin alternative because it forms a thermally irreversible gel and requires a low pH and high soluble solids content. Carrageenan, on the other hand, is used for chewing gum-type or molded candies [1]. In gelatin-based inks, the addition of small amounts of kappa-carrageenan (κ-carrageenan) or gellan gum creates protein–polysaccharide co-networks that increase early yield stress and enhance storage modulus at build temperature. These formulations enable extrusion at practical pressures and produce cleaner filaments with improved wire perpendicularity and wall angle retention. In systems based on fish gelatin and high-noble gellan gum, comparable performance is achieved at modest total solids amounts, consistent with clean-label goals in three-dimensional food printing [16].

When gelatin is purified, it exhibits low immunogenicity, thereby improving drug delivery and microstructural homogeneity compared to alginate, and provides superior structural integrity compared to starch and cellulose, making it highly suitable for oral, parenteral, and implantable drug delivery systems, unlike some polysaccharide carriers. Alginate, an anionic polysaccharide derived from brown algae, is preferred due to its mild ion-mediated gelation, which allows for the encapsulation of cells and sensitive therapeutics. Cellulose and its derivatives are widely used in oral controlled-release systems due to their stability and adjustable solubility. Guar and xanthan gum are low-cost, highly swelling, and biodegradable hydrocolloids, but their poor mechanical strength limits their use in advanced pharmaceutical applications. Biopolymers such as chitin, alginate, starch, cellulose, and gums offer certain advantages, but their applications are generally limited by solubility, mechanical, and reproducibility issues. In contrast, gelatin offers a multifunctional and balanced profile by combining enzymatic degradability, intrinsic bioactivity, tunable mechanical/physicochemical properties, and versatile processability. Gelatin is less immunogenic and water-soluble, making it easier to process into various drug delivery formulations (e.g., nanoparticles, hydrogels) compared to its precursor with a rigid triple helix structure (such as collagen) [17].

## 2. Structure, Chemistry, and Physicochemical Properties of Gelatin

**Figure 3:**
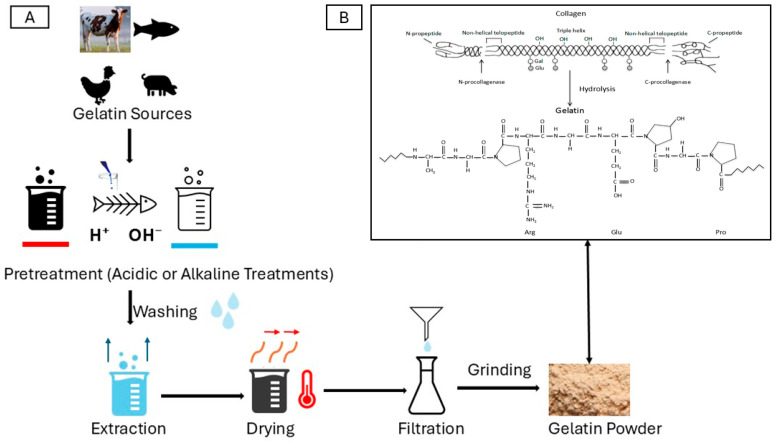
(**A**) Process flow chart in gelatin production; (**B**) collagen hydrolysis to gelatin schematic structure [14].

Gelatin, abundant in mammalian tissues, is derived from collagen. Collagen is the main component of connective tissues, bones and cartilage, tendons, skin, and scales (Figure 3). It consists of three chains and has a molecular weight of 350 kDa. Each chain has a molecular weight of 105 kDa and a length of 300 mm. Gelatin is obtained by partial hydrolysis and cross-linking of collagen [18,19]. The hydrolytic transformation of collagen causes the polypeptide chains to break, and the molecular weight of gelatin varies within these polypeptide chains. Depending on the gelatin production process, different chain types are called single α-chains, β-chains, and γ-chains, with molecular weights ranging from 80 to 125 kDa, 160 to 250 kDa, and 240 to 375 kDa, respectively [20,21,22]. The process of obtaining gelatin from collagen consists of three main stages: the first stage is washing the collagen source and subjecting it to alkaline, acidic, or enzymatic pre-treatment. The pre-treatment is determined according to the collagen source. Acidic treatment is applied to raw materials with fewer covalent cross-links, such as fish and pork, while alkaline treatment is applied to bovine hide, which contains more covalent bonds and has a complex structure. These processing differences result in the formation of different types of gelatin, such as gelatin A and gelatin B. The second stage is the extraction step, where heat is applied. The third stage involves filtering, drying, and powdering the gelatin [18,19]. Gelatin A and gelatin B are two types of commercial gelatin. Gelatin A is a cationic gelatin and is obtained by partial acid hydrolysis of collagen. During this process, the amide groups of glutamine and asparagine are converted into carboxyl groups, thus increasing the isoelectric point of the protein (pI = 7–9). Gelatin B is anionic gelatin obtained from alkali-based processing of collagen. During alkaline hydrolysis, the amide groups of asparagine and glutamine are partially removed, and an increase in aspartic and glutamic acid content occurs. As a result, the increase in carboxyl groups causes gelatin B to become negatively charged, thus giving it a lower isoelectric point (pI = 4.7–5.5). Accordingly, depending on the type and parameters of the extraction process, gelatin can have different isoelectric points depending on the degree of separation of free carboxyl and amino groups. In general, gelatin, like collagen, is characterized by the repetition of the (Gly-XY)^n^ triplet. One-third of the chain consists of glycine, and one-third consists of proline or hydroxyproline. Gelatin has cationic, anionic, and hydrophobic groups in approximately a 1:1:1 ratio. Therefore, approximately 13% of the polypeptide chain of gelatin consists of lysine and arginine, approximately 12% consists of negatively charged glutamic and aspartic acid amino acids, and approximately 11% consists of hydrophobic leucine, isoleucine, methionine, and valine [23]. Since gelatin mostly contains denatured collagen, its amino acid composition is like that of collagen molecules, but there are differences in the production method. For example, alkaline processing converts glutamine to glutamic acid and asparagine to aspartic acid, resulting in a higher ratio of aspartic acid to glutamic acid in type B gelatin compared to type A gelatin [24].

Furthermore, controlling the temperature during processing allows the triple helix collagen to be broken down into gelatins with different molecular weights, enabling production with varying degrees of mechanical strength. Due to its excellent biocompatibility, biodegradability, low toxicity, and low allergenicity, triple helix collagen has emerged as an ideal choice for bone tissue scaffold materials [25]. Gelatin is a protein fraction that does not occur naturally but is obtained after processes such as cross-linking and polypeptide bond cleavage between collagen polypeptide chains. Global production of gelatin derived from pig skin is higher than that of other types, accounting for 46% of total gelatin production, followed by bovine skin gelatin at 29.4% and bovine bone gelatin at 23.1% [19]. Fish gelatin (50–150 g) has lower gel strength, a lower melting point (~25–30 °C/35–40 °C), and lower proline and hydroxyproline content (10–15%/20–25%) compared to bovine and porcine gelatin (200–300 g), resulting in lower thermal and mechanical stability [15] (Table 1). In the cosmetics industry, it is used in hair gels, creams, and dyes. In the biomedical field, it is used in natural activities such as antidiabetic and anticancer activities. In the pharmaceutical industry, it is used as a gelling agent. In photography, it is used in films that preserve the life of photographs, and its applications are also widespread in the fertilizer and paint industries [19].

It is clear how the conformational organization of gelatin differs from highly organized natural collagen depending on the degree of collagen denaturation. Advanced techniques determine the degree of collagen denaturation; X-ray diffraction has shown a certain degree of fibril-like structures in gelatin, but these structures are not comparable to well-organized collagen networks. Depending on the gelatin concentration and the temperature and energy required for the formation of secondary structures, the polypeptide chains of gelatin can have different spatial arrangements and thus different interactions during gelation [23].

Gelatin is used in many different industries due to its low cost, easy availability, gelling structure, and elasticity, but it has certain limitations regarding heat, temperature, etc., as well as some dietary and religious restrictions (Table 2) [26].

Several parameters affect the use and quality of gelatin, such as solubility, viscosity, melting point, and solidification point [1]. Solubility describes gelatin swells and increases in volume upon contact with cold water; when heated above its melting point, hydrated gelatin decomposes and passes into solution, forming a gel when cooled, and is insoluble in non-polar solvents such as alcohol, mannitol, and glycerin. The viscosity produced by gelatin solutions imparts functional properties to the samples. The melting point is the temperature at which the gelatin gel softens sufficiently and allows carbon tetrachloride droplets to pass through it, and the solidification point varies depending on thermal and mechanical background, but higher solidification temperatures are obtained when the gelatin solution is cooled slowly compared to rapid cooling. Mechanical action inhibits or delays solidification [1].

## 3. Gelatin-Based 3D Printing Technologies

Three-dimensional printing technology, with its superior manufacturing capabilities, is used in many fields such as biomedical, automotive, aerospace, electronics, construction, food and health, and sustainable energy [29]. In gelatin products produced using traditional methods, the material is obtained in fixed and specific flat layer-like forms, and the porosity and density distribution of the product are not homogeneous. However, 3D printing with computer-aided design allows for the creation of a homogeneous and precise distribution in the product by building the material layer by layer. One of the best examples of this is the production of tissue scaffolds with specific pore sizes to which cells can adhere, and marine gelatin is used in functional food production to obtain different textures. In this way, the biocompatible and functional properties of gelatin are used more effectively, its application areas are expanded, and the development of complex structures that cannot be produced with traditional methods is enabled. Traditional production, unlike 3D printing, involves the piecemeal production and assembly of parts, and includes some tool accessibility limitations. Due to these limitations, traditional manufacturing is insufficient. However, 3D printing allows to produce more complex structures with less weight, resulting in a more efficient, innovative, high-performance manufacturing technology that creates complex structures [5]. Three-dimensional printing can alleviate organ shortages and the need for organ transplants by enabling in vivo transplants; three-dimensional-printed chips can combine microfluidic procedures in a single place by controlling chemical and biological processes in a lab environment; and microvascular networks can also be produced [29]. In 2020, the gelatin market was worth USD 3.18 billion, and in 2013, the highest share of the gelatin market was used in the food and beverage sector (28%), followed by nutritional supplements (25.8%), pharmaceuticals (21%), and cosmetics (5.5%) [30]. Given its widespread use, the inclusion of gelatin in 3D printing processes is valuable. Gelatin’s gel structure, viscosity, easy availability, and biocompatibility make it suitable for 3D printing. Among the various valuable products obtained from waste, collagen and gelatin extraction is an ideal way to utilize waste due to its effective applications in the biomedical and pharmaceutical industries [31]. Table 3 summarizes some works related to gelatin-based applications in 3D printing.

There are studies on 3D printing of gelatin-based materials, and they have mostly used gelatin in combination with different hydrocolloids. Commonly used 3D printing techniques are extrusion-based printing, inkjet printing, and binder spraying. Extrusion-based printing is a method of producing food models by passing food products through a nozzle under constant pressure, where the food is continuously pushed through the nozzle under pressure, creating a continuous print. Furthermore, extrusion-based 3D printing is considered the best option for printing thermoplastic materials [40]. The inkjet printing (IJP) technique is the deposition of liquid materials or solid suspensions, which is a component of the low-temperature, low-pressure process. This method can use polymer-based deposition and dielectric and conductive nanoparticles, making it applicable to a wide variety of materials. It is also commonly used for chocolate, wet pastries, sugar cream, pork tenderloin, cheese, jams, and gels [40], and in binder spraying technology to create 3D prints, where powder particles are bonded and condensed in a powder bed using a liquid binder. Binder spray 3D printing offers rapid production, the capacity to structure objects using flexible material compositions, and the ability to produce products with high solid content [40]. For example, Singh et al. used different ratios of cellulose nanocrystals and gelatin to prepare and characterize scaffold structures using ink writing techniques. The scaffold structures were found to have a porous microstructure with a high swelling rate between 390 and 590, a porosity of 65%, no toxicity, and high biocompatibility, determined through in vitro biocompatibility tests, proliferation, and hemolysis tests. The optimum formulation was functionalized with nitric oxide-releasing modified gelatin to increase the applicability of the scaffold, so the functionalized scaffold was determined to have the potential to be used as a wound healing material through biocompatibility, cell proliferation, hemocompatibility, and cell migration studies [27]. In another study, Liu et al. produced 3D-bioprinted hydrogel patches containing nanomedical material for personalized cancer treatment in an implantable form. The main ingredient is fish-derived gelatin methacryloyl (F-GelMA), semi-synthesized fish gelatin methacryloyl, obtained from cold-processed fish gelatin. The low melting point and viscosity of F-GelMA were significantly improved by the addition of carboxymethyl cellulose (CMC), and the increase in particle size was prevented by the addition of CMC [41]. Similarly, Zhang et al. focused on and successfully produced double-network interwoven hydrogels consisting of alginate cross-linking and f-GelMA cross-linking to expand the application of fish gelatin in tissue engineering. In the presence of the f-GelMA network, the alginate/f-GelMA hydrogel exhibited significant properties compared to pure alginate and f-GelMA hydrogels, especially in the areas of mechanical strength, swelling rate, degradation, and cell behavior. As a result of this study, the use of gelatin obtained from fish or other marine products provides an alternative to mammalian gelatin for further research in 3D bioprinting [42]. All in all, these works successfully show the use of fish gelatin used for tissue engineering safely.

Palamidi et al. worked in the field of developing biocompatible printable inks based on chitosan, collagen, and gelatin, and continued their research for 3D printing. The printability and final product quality of various chitosan–gelatin (CS-Gel) hydrogel inks were evaluated. The inks were extruded at an extrusion pressure of 150 ± 40 MPa for optimum printing. Inks with low chitosan concentration (<4% *w*/*v*) showed poor printability, while inks containing 4% *w*/*v* chitosan and 1% *w*/*v* gelatin (CG) exhibited satisfactory extrusion and print quality. The results show that the developed hydrogel inks and optimized printing parameters can produce suitable scaffolds for tissue engineering applications. Finally, the cell compatibility of the 3D-printed structures was confirmed on fibroblasts by MTT assay, and the antimicrobial activity of the drug-loaded structures was tested against *E. coli* and *S. aureus*; in the presence of the KOH-treated CG Levo-printed structure, it was shown that the bacteria-free region increased from 8 ± 0.4 mm in the control group to 16.4 ± 0.37 mm against *E. coli*. The 3D printing of the structures: 3D printing was performed with an extrusion-based 3D bioprinter using stainless steel needle nozzles. A rectangular STL file with dimensions of 20 × 20 × 1 mm was used for 3D printing, and slicing of the STL sample was completed with Slic3r software (Rome, Italy). The height of the first layer was 0.2 mm, and the height of the other layers was 0.1 mm, with an angle of 90° between the layers. The printing pressure was at the minimum pressure required to create a continuous support rod for each hydrogel, and the printing speed was 5 mm/min. Ink cartridges were heated to 37 °C. Print quality was evaluated, and the optimal conditions for extrusion capability and print resolution were determined. Improved results were observed with the selected ink composition, CG (containing 4% chitosan, 1% gelatin, and 0.1% collagen by weight), especially when cross-linked with KOH. This highlights the usability of 3D-printed structures for various biomedical applications, particularly in wound healing and drug delivery. This study evaluates the 3D printing performance of different ink mix designs and describes the printing conditions; however, the fact that 3D printing conditions are specific to a particular printer or environment may limit the generalizability of the results [43]. Mekonnen et al. instantly produced PLA/gelatin nanofiber membranes for in situ skin defect repair in both in vitro and in vivo environments using a handheld electrospinning device, which they designed and manufactured with a 3D printer. Experiments showed that PLA/gelatin has superior material properties and biocompatibility, confirming that it is an excellent material for skin repair and can be used in the future for repairing skin defects [44]. However, hydrogels are attracting great interest in the biomedical field for their use in 3D printing, both as scaffolds and for the design of new medical hydrogels [45]. In general, 3D printing inks need to meet the following requirements: printable; biocompatible; suitable mechanical properties; good degradation kinetics; safe degradation by-products; and tissue biomimicry requirements [46].

## 4. Rheology and Printability

Hydrogels are network-structured polymers with high water-carrying capacity, resembling the extracellular matrix. While traditional methods disregard the porosity and density of the material in manufactured products, and the material is formed in fixed and specific flat layers, 3D printing, as additive manufacturing, allows for a homogeneous and precise distribution of the material by forming it layer by layer. Biopolymers used in hydrogel synthesis primarily include polysaccharides and proteins. Commonly used polysaccharides include alginate, chitosan, hyaluronic acid, and pectin. Collagen, gelatin, and silk fibroin are examples of frequently prepared protein-based hydrogels. These hydrogels can be used as drug delivery systems, scaffolds for tissue engineering, wound dressings, and bioinks. The physicochemical properties of hydrogels determine their suitability for 3D printing using different techniques. Two key factors determining printability are rheological properties and gelation mechanisms. For example, high-viscosity hydrogels have shown high printing accuracy. The flow behavior of hydrogels, i.e., their rheological properties across nozzles, determines their suitability for 3D printing. These properties are primarily categorized as viscosity, shear thinning, and yield strength. Low viscosity during printing and sufficient mechanical strength afterward are the ideal conditions to achieve. Rheological experiments are conducted to evaluate the suitability of the proposed hydrogel for 3D printing. Although lower viscosity is preferred for 3D printing, very low viscosity causes problems related to fluid flow. A high G′ (storage modulus) value is required for shear thinning behavior to occur. When comparing G′ (storage modulus) and G″ (loss modulus), the criterion G′ > G″ is valid for gelation to occur. To improve the printability of hydrogels, materials with faster recovery properties would be more suitable. The relationships between print head speed, volumetric flow rate of material passing through the nozzle, and shear rate are influenced by microstructural properties that vary depending on the ratios and materials used and by whether the cross-links between polymers are reversible or not [47]. Rheological properties such as gel strength and viscosity, as well as thermal stability properties such as melting and gelling degree, and fundamental physicochemical properties such as solubility, color, transparency, taste, and odor, determine the quality of gelatin. Gel strength is defined by a value called the “Bloom value,” and this value has been determined to be optimum between pH 5 and 9. The viscosity of gelatin is dependent on concentration, temperature, and pH, and it has been found that viscosity increases with polymer concentration and decreases with temperature and pH. The thermal stability of gelatin is affected by polymer concentration, molecular weight distribution, and Bloom value parameters. The viscosity and Bloom strength of gelatin are affected by its molecular weight. The viscosity of the liquid determines the movement of gelatin molecules, while Bloom strength measures its ability to form a gel. The amount of the triple helix structure present determines the Bloom strength, typically ranging from 30 to 300 g. A Bloom value below 150 is considered low-grade, between 150 and 220 medium, and between 220 and 300 high. High Bloom values indicate high molecular weight, high viscosity, high strength, high melting and gelling points, and rapid gelling time. Gelatin obtained from mammals has lower gelling temperatures and Bloom strengths than fish gelatins [28].

However, the melting point of mammalian gelatin is generally in the range of 28–31 °C, while for fish-derived gelatin it is in the range of 11–28 °C. Furthermore, it has been shown that the gel strength, gelation point, and melting point of mammalian gelatins are higher than those of fish-derived gelatins. Indeed, typical gel strength, gelation point, and melting point temperatures for mammalian gelatins are in the range of 100–300 Bloom, 20–25 °C, and 28–31 °C, respectively, while the values for fish gelatins are approximately 70–270 Bloom, 8–25 °C, and 11–28 °C, respectively [23].

**Figure 4:**
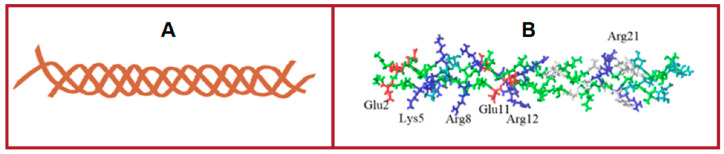
Gelatin fragment structure: (**A**) triple helix structure of gelatin; (**B**) positively charged residues are blue, negatively charged residues are red, polar residues are green, and hydrophobic residues are gray [48].

It is understood that when gelatin is exposed to low temperatures in the gelation protocol, the gelatin chains undergo a conformational transition from disordered to ordered and can form thermally reversible networks through the formation of hydrogen bonds. Gelatin is used in the food industry, particularly as a thickener in confectionery and jams, a clarifying agent in wine, beer, and fruit juices, an emulsifier in sweets, a stabilizer in ice cream and cheese, a texture regulator, and a film-forming agent in coatings for meats and confectionery. In the cosmetics industry, it is used in face creams, body lotions, shampoos, hair sprays, sunscreens, and bath salts due to its moisturizing effect (Figure 4) [23].

### 4.1. Rheological Characterization

Viscosity is the most important rheological property, and a decrease in viscosity with increasing shear rate is crucial because shear thinning is necessary for easy extrusion of inks. Flow curves are obtained by varying the shear rate or shear stress, and the corresponding apparent viscosities are measured. In extrusion printing, if the nozzle is moved by pneumatic pressure, the flow curve measured by varying the shear stress is used. If the nozzle is moved by a piston, the flow curve obtained by varying the shear rate is more suitable. Generally, hydrogel inks exhibit a constant viscosity at low shear rates, which is called zero shear viscosity; subsequently, a decrease in viscosity occurs with increasing shear deformation, and finally, a constant viscosity occurs again at high shear deformations. Viscosity-shear rate data can be fitted to the Carreau–Yasuda and Cross models. For printing applications, the shear thinning region in the viscosity-shear rate data is fitted to the Ostwald–de Waele power law model [49]. Yield stress is the minimum force required to initiate the flow of the ink and is determined by the shear stress ramp test. The shear stress is plotted against the shear rate, and the data are fitted to the Herschel–Bulkley model to determine the yield stress. Post-shear recovery is defined as the period after shear thinning in the extruder, during which the ink must solidify and settle on the print bed upon exiting the nozzle. Therefore, the ink must regain its viscosity and structural strength. Rheologically, the G′ value of the inks should be greater than G″, representing high structural strength. Ink recovery can be evaluated with both constant shear and oscillatory deformations. In these measurements, the ink is subjected to low–high–low deformation cycles [49]. To be considered suitable for bioprinting, bioinks must possess appropriate mechanical, rheological, chemical, and biological properties. The viscoelastic properties of bioinks must be carefully controlled when designing them for extrusion-based 3D bioprinting to ensure they flow easily through the printing nozzle and retain their shape and structure after deposition. Achieving this requires a comprehensive understanding of the interactions between printing parameters (such as extrusion pressure, printing speed, and nozzle size) and the rheological properties of the material (such as viscosity, shear modulus, and elasticity). Typically, additives such as gellan gum, hyaluronan, or carrageenan increase the yield stress of a given ink. In the presence of a positive ion in solution, gellan gum is added to gelatin, methacryloyl, forming a shear-reversible, ionic cross-linked network, which increases the viscosity of the ink at rest. Shear forces damage this reversible network during deployment, and the network repairs itself when the shear forces cease [10].

Rheological properties of 3D printing inks are critical determinants of extrusion capability in extrusion-based 3D printing. As shear stress near the nozzle increases, the viscosity (η) of the ink changes under shear, affecting the extrusion behavior. Consequently, η is an important metric for evaluating the suitability of the ink for additive manufacturing [7].

The Poiseuille equation is derived for Newtonian fluids under steady laminar flow conditions using the conservation of momentum (Equation (1)):

(1) $$p(\pi r^{2})-(p+∆p)\pi r^{2}=2\tau \pi rL$$

where p represents pressure, τ represents shear stress, and L represents length. The nozzle shear stress profile is obtained as follows (Equation (2)):

(2) $$\tau =\frac{-∆p}{2L}\;r$$

In this way, the behavior of hydrogels and their suitability for 3D printing are determined by calculating the shear stress values during the printing process, and the polymeric solution is usually non-Newtonian. Therefore, the power law model expressing the shear stress is as follows (Equation (3)):

(3) $$\tau \;=K(\dot{\gamma })n$$

Here, K represents the flow consistency index, n represents the flow behavior index, and both parameters are determined from experimental curve data correlated with shear stress $\dot{\gamma }$. For Newtonian fluids, the value of n is 1, and for shear-reducing fluids, the value of n is less than 1. The values of K and n indicate the microstructural properties of the solution and vary depending on concentration, crystallinity, and molecular weight [47].

The values vary depending on the shear rate used and the combination of other polymers, but in any case, the apparent viscosity of a power-law fluid is compared with Newton’s law, which gives the final form of the shear rate, using the following formula (Equation (4)):

(4) $$µ={K(\dot{\gamma })}^{n-1}$$

Non-Newtonian fluid can be expressed as follows (Equation (5)):

(5) $$\dot{V}=\pi R^{2}V\;=\;\pi (\frac{n}{3n+1})(\frac{-∆p}{2mL}{)}^{1/n}R^{\left(\left(3n+1\right)/n\right)}$$

where $\dot{V}$ describes volumetric flow rate.

Next, assuming the hydrogel volume remains constant before and after printing, the water ratios are determined as follows (Equation (6)):

(6) $$\frac{\dot{\gamma }2}{\dot{\gamma }1}\;=\;{\left(\frac{D1}{D2}\;\right)}^{\left(3+\frac{1}{n}\right)}$$

where $\dot{\gamma }2$ represents shear rate at syringe and $\dot{\gamma }1$ represents shear rate at nozzle, and (Equation (7))

(7) $$V_{2}=V_{1}{\left(\frac{D1}{D2}\right)}^{2}$$

V_2_ represents the piston speed, and V_1_ represents the speed of the extrusion of hydrogel, D1 and D2 are the inner diameter of the syringe and the inner diameter of the nozzle, respectively [47].

Filling characteristics contribute to print quality by minimizing the effects of gravity. For example, if the hydrogel has a high gelatin rate, a tendency to produce a high-quality print is observed because high viscosity prevents the hydrogel from spreading after placement, and rapid gelation quickly solidifies the hydrogel, preventing print deformation [47].

The material requirements for gel food printing by 3D printing were determined based on rheological parameters such as storage modulus (G′), yield strength (τ0), and angle (δ), as well as accuracy, shape retention, and extrusion capability. In this study, 3D printing materials were produced with gelatin-based formulations containing gelatin, gum, and pectin. While formulations containing pectin yielded higher δ and lower G′ and τ0 values, gum mixture-based formulations formed a gel with higher G′ and δ values and a wider τ0 range [50]. For a printable biopolymeric ink, it must have viscoelastic and thixotropic properties so that the ink flows easily from the nozzle. The flow property of the ink is an indicator of quality in the final product and is necessary for the optimization of the final product, determining the material selection required for product optimization. To prevent deformation in the final product for 3D printing, bio-based polymeric inks can be used to develop stable polymeric networks that exhibit limited shrinkage during drying and resist stress caused by capillary forces [51]. The efficient and sustainable utilization of food waste is important, but ensuring its proper implementation is a challenge. Carvajal-Mena et al. used 3D printing technology to develop a suitable material based on salmon industry by-products and evaluated the rheological, textural, and printability of salmon skin gelatin gels (SGG) at concentrations of 2%, 5%, 8%, 11%, and 14%. According to the results obtained, salmon gelatin gels at different concentrations showed non-Newtonian shear thinning, thixotropy, and viscoelastic behavior with a dominant elastic component (G′ > G″), revealing the material’s ability to flow as an independent filament and form 3D structures. It was observed that SGGs exhibited shear thinning and viscoelastic behavior with a dominant elastic component, and textural property analysis and printability analysis determined that 8% SGG could be easily extruded and form stable structures after printing. The most suitable biomaterial for extrusion-based 3D printing is 8% salmon gelatin. It was designated as SGG because it conformed to the 3D models in the final printed product and maintained its dimensional stability over time [52]. Cheng et al. investigated the effect of phase morphological transformations in a corn starch/gelatin system induced by changing the gelatin content on rheological properties and 3D printing performance. Added gelatin prevented the gelatinization and retrogradation of the starch, causing a transformation of the continuous phase structure. The transition process from the starch continuous phase to the gelatin continuous phase resulted in a thinner gel wall structure, relaxing the dense network and causing a decrease in flow stress (τf) and storage modulus (G′). The printed 3D model is a rectangular prism with a height of 15 mm, a width of 30 mm, and a length of 30 mm. The extrusion temperature was set to 25 °C, while the 3D printer’s movement speed, retraction speed, and filling speed were determined as 25 mm/s, 40 mm/s, and 30 mm/s, respectively. The nozzle diameter used was 0.84 mm. It was determined that the bonding temperature (Ptemp) and peak viscosity (PV) values were affected by factors such as the swelling degree of the particles, water-binding capacity, shear resistance, and the competition of free water between the unhydrolyzed starch granules and the filtered amylose. While the Ptemp value of the starch increased with the addition of gelatin, the PV value decreased. This also shows that the addition of gelatin increased the anti-swelling property of starch granules. These findings reveal that gelatin is an excellent additive in the development of ready-to-print starch-based food printing inks and is extremely important in 3D printing for the creation of flour-based products [53].

All in all, the performance of gelatin in 3D printing applications is largely influenced by its rheological properties mentioned above. This rheological characterization is also important for process optimization of the printing process and for evaluating the suitability of bioink for use in bioprinting. In this context, the use of gelatin as bioink and the evaluation of the necessary sterilization process in bioprinting applications are of great importance.

### 4.2. Stabilization of Bioink

Sterilization is critical in bioink and 3D bioprinting processes to prevent contamination, ensuring cell viability, product safety, and the accuracy of experimental results. The most common process is the autoclave process, which occurs under high temperature and pressure, typically at 121 °C for 15 to 30 min. It leads to microbial death and reduces the printability and viscosity of bioink. While widely used and effective, the high temperature requirement negatively impacts nutritional value [54]. Membrane filtration typically uses a 0.22 µm filter and is effective against many microorganisms. It reduces the viscosity and yield stress of bioink and does not affect its gel structure. It is ideal for liquids and preserves material integrity, but small pore sizes can lead to bursting [54]. Ultraviolet ray application causes microbial irritation and reduces the viscosity and yield stress of bioink. Furthermore, although it is cost-effective, its efficiency varies depending on the type of microorganism and is not stable [54].

**Figure 5:**
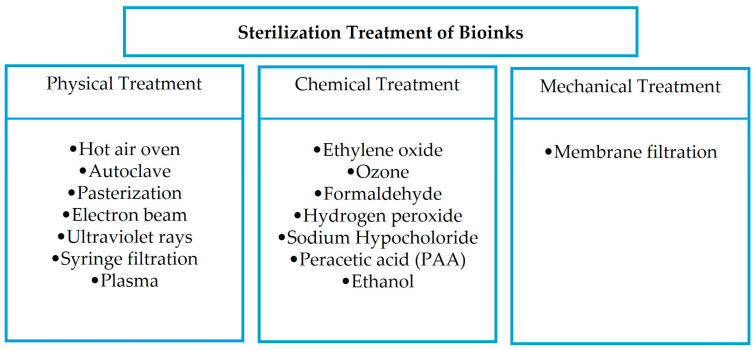
Sterilization methods of bioink [54,55].

The main methods used in bioink sterilization in industry and academia are summarized in Figure 5.

Successful 3D food printing depends not only on the printing technology but also on the properties of the food material or “ink.” Together, these factors determine whether a formulation can be reliably printed, maintain its structure, and effectively deliver bioactive compounds [56].

### 4.3. Factors Affecting Printing Quality and Stability

One of the important parameters is print speed and resolution; higher speeds can speed up production but can lead to uneven extrusion or layer placement if the material hardens too quickly. Fine nozzles provide higher resolution but require more pressure and carry a risk of clogging, especially when dealing with antioxidant particles or viscous mixtures. Resolution is generally in the millimeter range for food printers, which is acceptable for most functional food purposes (shapes, layered internal structures) but not as fine as non-food 3D printing [56]. Another one is nozzle temperature, and in extrusion printers, the nozzle or food reservoir is sometimes heated (a typical temperature for chocolate is ~30 °C, just above its melting point). For other foods, printing is completed at room temperature or slightly warm temperatures. Temperature control is vital; for example, meat paste may need to be kept cold to maintain its consistency, while a gelatin-based ink needs temperature to flow and then cooling to gel. Another factor is particle size and texture; for smooth printing (especially from ~1 mm nozzles), solids or fibers should be finely ground or pureed in the food ink. Large particles, such as nut pieces and coarse grains, can cause clogging. Adhesiveness and drying behavior: Printed layers should adhere to each other but not spread. Controlling moisture content is crucial—too much water can cause the structure to collapse; too little water can prevent the material from flowing. During or after printing, some products are dried or baked to fix the structure; for example, a printed cookie might be baked to achieve a crispy texture. Materials that gel when cooled or when cross-linkers are added (such as alginate which hardens with calcium ions) are useful [56].

### 4.4. Cross-Linking Mechanisms and Their Influence on Gelatin Performance

**Figure 6:**
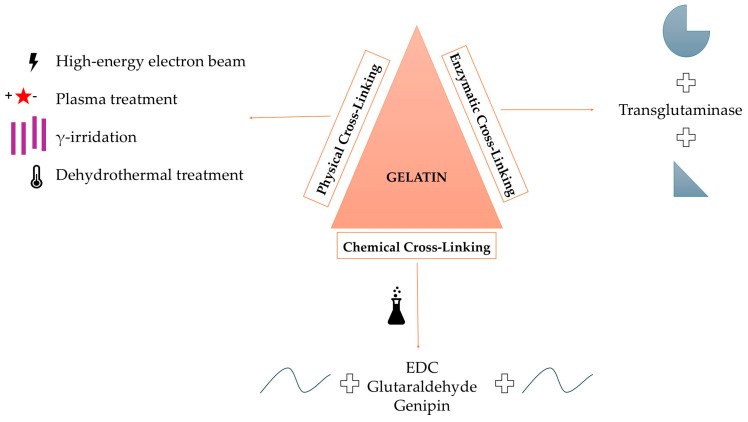
Crosslinking methods main categories: physical, chemical, and enzymatic methods.

Cross-linking plays a critical role in determining the degradation kinetics, mechanical performance, and rheological properties of gelatin. In 3D printing applications, cross-linking can be applied pre-printing, during printing, or post-printing, depending on the intended function. Pre-printing cross-linking is generally used to adjust the viscosity and printability of gelatin-based formulations, while post-printing cross-linking is mostly preferred to increase structural integrity, mechanical strength, and resistance to degradation. In some cases, in situ cross-linking, which occurs simultaneously during the printing process, provides immediate stabilization of the deposited layers. Cross-linking methods are generally divided into three main categories: physical, chemical, and enzymatic methods (Figure 6). Physical methods include high-energy electron beam, plasma treatment, γ-irridation, and dehydrothermal treatment. Examples of chemical methods include 1-Ethyl-3-(3-dimethylaminopropyl) carbodiimide (EDC), glutaraldehyde, and genipin. Enzymatic methods include microbial transglutaminase [57]. However, increasing mechanical strength and ensuring stability are essential, especially in biomedical applications [58]. Various cross-linking methods exist to achieve these mechanical properties in gelatin. In physical cross-linking of gelatin, covalent bonds are formed between the polymeric chains of gelatin. The necessary energy can be obtained using an electron beam, and the cleavage occurs through the formation of free radicals, creating bonds between the gelatin polymeric chains. In the plasma method, hydroxyls are formed after the reaction of oxygen radicals with water, and in this way, cross-links are formed between the gelatin polymeric chains. Dehydrothermal (DHT) cross-linking is performed under vacuum at high temperature and low pressure. This combination causes water to condense and separate from the gelatin, thus promoting cross-link formation [57]. Chemical cross-linking leads to the formation of covalent bonds between the polymeric chains of gelatin, resulting in stable gelatin hydrogels with controllable physical and mechanical properties [57]. One of the most used cross-linkers is glutaraldehyde, which reacts rapidly with the amino groups of gelatin, is relatively inexpensive and easy to use, and is highly soluble in aqueous solution. 1-Ethyl-3-(3-dimethylaminopropyl) carbodiimide (EDC) is also used in gelatin cross-linking, and carbodiimides, which are allene-structured unsaturated compounds, are non-toxic and biocompatible. Reducing sugars such as glucose and fructose can also be used as cross-linkers in gelatin, increasing the yield of cross-linked products; they are non-toxic and inexpensive. Glucose can also react and polymerize at high temperatures. Another cross-linker is the naturally occurring genipin, which has low toxicity, high biocompatibility, and self-polymerization capabilities. It increases the thermal and structural stability of gelatin. Genipin covalently binds to the amino acids present in gelatin, usually lysine and, to a lesser extent, hydroxylysine and arginine, via an SN2 nucleophilic substitution reaction. Another cross-linking agent is the hexamethylene diisocyanate (HMDC) molecule, which is insoluble in water and highly reactive in aqueous solution; therefore, a surfactant is used in the cross-linking reaction, even though it is quite expensive, but biocompatible [57,59]. Transglutaminase, used as an enzymatic method, is employed to promote the formation of covalent cross-links between gelatin chains. The transglutaminase enzyme catalyzes the acyl transferase reaction between glutamine residues and primary amino groups of gelatin [57]. The choice of the appropriate cross-linking method depends on the intended application but requires an evaluation of mechanical strength, degradation rate, and biocompatibility. The physicochemical and structural properties of bioinks prepared from different animal-derived gelatins were evaluated, highlighting the influence of gelatin type and concentration on pH, color, viscosity, and printability. Pork gelatin exhibited superior viscosity and was the only type capable of forming stable 3D scaffolds. Notably, 20% pork gelatin, when blended with alginate (1:1 ratio, 6% *w*/*v*) and cross-linked using 100 mM CaCl_2_, demonstrated enhanced gel strength and structural integrity, indicating its suitability for 3D bioink applications [60]. Another researcher described making a 3D-printed scaffold based on cold-water fish skin gelatin and alginate, which demonstrated enhanced mechanical strength and biocompatibility through double cross-linking. The scaffold supported cartilage regeneration in vivo, indicating its potential for tissue engineering applications [61].

A recent study demonstrated that transglutaminase (TG) cross-linking of porcine skin gelatin significantly enhanced the molecular structure, thermal stability, and rheological properties of gelatin-based high internal phase emulsions (HIPEs). Prolonged cross-linking improved viscosity, structural integrity, and resistance to high-temperature processing, enabling stable storage. These improvements also enhanced 3D printability, highlighting the potential of TG-cross-linked porcine gelatin HIPEs in food applications [62].

Another study found that gelatin-based composite hydrogels (35% gelatin, 5% glycerol) cross-linked with transglutaminase (5%) exhibited improved structural stability and 3D printability using heated extrusion. The formulation maintained good formability even with the addition of 15% brown sugar, enabling nutrient incorporation. These results highlight the potential of TG-cross-linked gelatin systems for customized and nutritionally enriched 3D-printed foods [63].

### 4.5. Importance of Post-Processing

Many printed foods are not ready to eat immediately after printing. Common post-processing methods include baking, cooking, drying, or freezing. Mild drying at low temperatures can intensify flavors and antioxidants without significant loss, while high-heat baking can reduce vitamin content. Therefore, printing and subsequent cooking processes should be considered together when evaluating nutrient preservation. For antioxidant-rich foods, low water activity can be a double-edged sword: it can slow microbial spoilage, but very dry conditions can sometimes promote oxidative reactions or reduce the effectiveness of certain antioxidants; for example, vitamin C is generally more stable at moderate moisture levels than in its fully dried state [56]. In biomedical applications, gelatin easily degrades under encapsulated drug form; post-processing is important for stability, so gelatin can be ensured by combining it with thermostable polymers [17].

## 5. Applications (Food, Biomedical, and Health)

In recent years, the applications of gelatin using 3D printing technologies have made a difference, particularly in the food, biomedical, and healthcare fields, and research into its unique physical and chemical properties and biocompatibility has become widespread. Especially with the advancement of materials science, the production of functional products is being enabled, and studies examining different application methods of these products have become concentrated in the literature [5,7].

Commonly used 3D printing technologies in biomedical applications include fused layered modeling (FDM), stereolithography (SLA), selective laser sintering (SLS), electron beam melting (EBM), and direct metal laser sintering (DMLS). FDM is cost-effective but insufficient for complex designs. SLA offers high resolution but is expensive and has limitations in print size. SLS, on the other hand, offers high precision and resolution but has surface quality issues. EBM offers high precision but has limited material selection. DMLS is precise and suitable for metal alloy use, but it is quite expensive and has limited print size [5]. In food production, 3D printing technology is mostly extrusion-based (Fused Deposition Modeling and Direct Ink Writing), SLS, SLA, and inkjet printing. Powder bonding technology is quite common in food production, and this process is based on the principle of bonding the powder with sugars and lipids in food products [51].

### 5.1. Biomedical Applications

The most common type of 3D printing for biomedical applications is bioprinting, which involves various additive manufacturing technologies to 3D print living cells, essentially depositing bioink in a layered manner. Three-dimensional printing modeling is particularly used in tissue engineering to create implants to replace damaged or dysfunctional tissues, and these implants need to be produced using biocompatible materials. Biocompatible materials must be suitable for the growth of living cells to support tissue growth. Implant and prosthesis design and production require materials science, engineering, pharmaceuticals, and long-term clinical monitoring. For complex structures such as bone regeneration, 3D printing supports the formation of complex structures. For example, one study comparing the response of 2D and 3D cultures of human colon cancer cell lines to treatment with the mTOR (mechanistic target of rapamycin) inhibitor rapamycin identified conflicting levels of protein kinase B mammalian target of rapamycin ribosomal protein S6 kinase (AKT/mTOR/S6K) signaling activity and concluded that the 3D microsphere model accurately represented the in vivo response to rapamycin treatment and created more accurate drug screening models compared to 2D cultures [64]. In recent years, the use of gelatin-based materials in biomedical and healthcare applications through 3D printing has become quite widespread due to its potential for personalization. In a study using poly (ε-caprolactone) (PCL) micronetworks and PCL/gelatin/ε-polylysine (ε-PL), easily produced PCL/gelatin/ε-PL nanofiber layers manufactured using electrospinning methods and scalable PCL/gelatin/ε-PL porous scaffolds are ideal for applications in skin wound repair. As a result, the produced scaffold exhibits uniform thickness, exhibits intermittent mechanical properties compared to human skin, and is suitable for use in wound dressings due to its hydrophilic properties. These findings suggest that scalable bimodal scaffolds have the potential to be effective wound dressings for tissue engineering and regenerative medicine [65]. Macrophage-supported microtissues were developed using GelMA as a macromer solution. It was noted that macrophages maintained viability after production, and mechanical properties could be controlled by GelMA concentration; thus, a strong effect on the proliferation and polarization of encapsulated cells was observed [66]. In this study, cell-loaded nanofibers were produced using a gelatin/pullulan mixture. The electrospinning properties of gelatin and the tensile strength of the pullulan mixture were enhanced, and the process was carried out with a voltage of 8 kV and a gelatin/pullulan concentration of 5 mg/mL. As a result, the adipose-derived stem cells (ADSCs) showed 90% viability, and the successful production of cell-loaded nanofibers using the gelatin/pullulan mixture was achieved [67]. In one study, alginate/gelatin hydrogel composites were designed to create aortic valves using an extruder-based 3D printer. After a 1-week culture procedure, cell density remained viable, and the addition of alginate ensured linear tensile-strain behavior in the hydrogel structure, resulting in high extensibility and mechanical strength [68]. GelMA is a fibrous membrane produced by 3D hydrogel and electrospinning methods and is used especially in biomedical fields. It is widely used in tissue regeneration. It can be used in different forms in 3D-printed scaffolds, and when applied to these scaffolds in combination with other polymers and small molecule drugs, it can meet the requirements for obtaining skin, tendons, bones, cartilage, vessels, etc. [69]. In the field of skin regeneration, GelMA, which is similar to natural dermal extracellular matrix (ECM) and has controlled mechanical and degradation properties, is used as the main material for use as a dressing [69]. In tendon regeneration, tendons connecting muscle and bone are subject to tearing and puncture due to load-bearing capacity. GelMA hydrogels can be used to fulfil the mechanical and biological requirements necessary for tendon regeneration [69]. Visser et al. strengthened the mechanical and biological structure by producing a GelMA composite microfiber mesh using 3D printing technology and reported that this composite structure showed similarity to natural tissue in terms of skeletal stiffness and elasticity [70]. In bone regeneration, mechanical shear force during injection causes low cell retention and damage to the cell membrane, hindering the formation of the structure for 3D printing [69].

### 5.2. Health Applications

Gelatin’s specific and unique structure makes it particularly suitable for use in regenerative medicine and tissue engineering. Various tissues and organs, including skin, blood vessels, and hearts, are frequently produced through 3D bioprinting (Figure S1 shows examples of gelatin-based 3D printing applications in biomedical industries and represents the fabrication process of a gelatin-based hydrogel organ created by 3D bioprinting technology, a gelatin/alginate/fibrin hydrogel mixture loaded with fat-derived stem cells, and a vascularized organ of β-cells under 3D bioprinting, as well as images of different types of fish skin gelatin scaffolds and SEM microstructure and temperature scans of mammalian and fish gelatin hydrogels [19,71,72]). These manufactured tissues and organs are used both as building blocks for the ultimate goal of repair and regeneration and as in vitro models for pharmacokinetics, drug screening, and other purposes [14]. Some studies on the use of gelatin in tissue engineering with 3D bioprinting: In research conducted on nerve tissue, gelatin methacrylamide was used in the SLA bioprinting process. Mouse neural stem cells were determined as the cell source, and according to the results obtained, it was determined that neural stem cells differentiated into neurons at the end of a two-week culture period, and it was reported that the printed structure showed neurite elongation. As a result, it was seen that neural structures created with 3D bioprinting have significant potential for the regeneration of nerve tissue [73]. In another study focusing on the liver, SLA bioprinting was performed using gelatin methacrylate, glycidyl methacrylate, and hyaluronic acid. Human-derived induced pluripotent stem cell-derived hepatic progenitor cells, human umbilical vein endothelial cells, and adipose (fat)-derived stem cells were selected as sources. As a result, it was determined that hiPSC-HPCs provided phenotypic and functional improvements in the 3D model [74]. In the field of 3D bioprinting, bioinks used to mimic tumor microenvironments are noteworthy. Some studies in this area include a microenvironment that was developed using mouse embryonic fibroblast cell line C3H/10T1/2 clone 8 cells, green fluorescent protein (GFP)-expressing human neonatal dermal fibroblasts, human umbilical vein endothelial cells, and red fluorescent protein (RFP)-labeled endothelial cells, targeting blood vessels in a tumor model. The bioink contains poly (ethylene glycol) diacrylate and methacrylate gelatin and was produced using 3D extrusion technology. The result was a vascularized and accessible tissue model [75]. In a similar study, a tumor model targeting blood vessels was created in a microenvironment of mouse epidermal fibroblasts using extrusion-based 3D printing technology, and the bioink used was gelatin-based alginate containing carbon nanotubes. As a result, the vascular structure was successfully formed with a hybrid hydrogel [76].

Suitable 3D structures can be created by using different hydrocolloids, and good results have been obtained in areas such as wound healing, dental, and limb implant design. Optimizing the 3D printing process for biomedical applications requires overcoming specific challenges to ensure reliable and high-quality production; design problems are overcome, particularly in medical implants, where complete elimination of pathogens is necessary, through appropriate material selection, sterilization, and proper surface passivation to prevent contamination. For example, polyethylene glycol (PEG) coating is effective in preventing protein adsorption [5]. Kuo et al. used gelatin and alginate (g/a) hybrid structures with 3%, 5%, and 7% concentration ratios and 1:2, 1:1, and 2:1 g/a ratios for extrusion-based 3D printing to produce bioskiescapes. Rheological properties were correlated with the 3D printing capability of hybrid hydrogel structures. The materials used for printing exhibited shear-thinning flow behavior and displayed a storage modulus (G′) higher than the loss modulus (G″) with a loss factor (tan δ = G″/G′) in the range of 0.48–0.58 in the frequency range of 15–40 rad/s. Tissue profile analysis revealed that among the most suitable formulas for 3D printing, the bioscaffold produced with 7% 1:2 G/hybrid gels had the highest stiffness and adhesion. The most common topic in bone tissue engineering is promoting bone repair, and gelatin is valuable for use in bone tissue engineering due to its biocompatibility [77]. However, the weak and single structural mechanical property of natural gelatin makes it unsuitable for use in cell culture alone. Therefore, a number of synthetic materials can be added to natural gelatin [25]. In another study, Zhao et al. applied a theory that encapsulated bone marrow-derived mesenchymal stem cells (BMSCs) and growth factors in GelMA microspheres using microfluidic-assisted technology. In this way, BMSCs survived, and bone formation was supported [78].

GelMA is a potential bioink for 3D printing and is used in tissue engineering for the production of skin replacement tissues [79]. Tanadchangsaeng et al. used fish skin-based GelMA bioink in 3D printing for skin replacement. They reported that cell viability increased in the 3D GelMA hydrogel scaffold and that biocompatibility was good, and in vivo results showed that adipose-derived stem cells supplemented with human platelet lysate (ASC + HPL) loaded GelMA scaffolds delayed wound shrinkage, especially at the wound edge, compared to the unadded sample group. The 3D bioprinting model was operated at a minimum pressure of 20–4 kPa, an inner diameter of 0.233 mm, a chronic nozzle diameter of 30 G, a printing nozzle speed of 5 mm/min, and a room temperature of 25 °C [79]. In another study, Maihemuti et al. created a 3D scaffold using cold-water fish skin gelatin to generate osteoarticular cartilage regeneration. They combined non-toxic and biodegradable cold-water fish gelatin with sodium alginate for viscosity and ease of compression, resulting in a hybrid hydrogel. The resulting scaffold mimics the original cartilage structure, facilitating nutrient transport and preventing further damage to the joint [61].

### 5.3. Food Applications

In food production, gelatin is utilized for its functional properties as a gelling agent, whipping agent, preservative colloid, binder, clarifying agent, film-forming agent, thickener, processing aid, emulsifier, stabilizer, and adhesive [1].

The suitable viscosity and gel-forming properties achieved in 3D printing result in stable printed structures (Figure S2—shows examples of gelatin-based 3D printing applications in the food industries and of marine-based gelatin Pickering gels and the 3D printing of marine-based residual-gelatin Pickering gels [16,80]). In milk-based printed foods, gelatin improves rheological properties and gel strength, enhancing post-printing shape accuracy and layer stability. In cases such as those requiring special dietary needs, it improves the texture and mouthfeel of the printed product, increasing consumer acceptability. A 3D food printer capable of producing natural food products is perceived as useful by consumers, and if the right strategy is provided, the likelihood of consumers deciding that a food printer is useful and having a positive attitude toward it increases. This positive attitude toward the printer increases the likelihood of purchasing one [81]. Due to the desire to ensure environmental sustainability while simultaneously providing high-quality and nutrient-rich foods, protein and polysaccharide-based edible films and coatings for food packaging attract interest as alternatives to traditional plastic-based packaging. Gelatin’s low cost, polymerization, biodegradability, and good antibacterial and antioxidant properties offer ideal possibilities for use in food packaging. Studies have been conducted by combining the obtained gelatin with other biopolymers to modify its mechanical properties and improve them [82]. Because the mechanical behavior of gelatin is very important, the texture of some products is affected by gelatin, and this affects the acceptance of the product by consumers. The results of rheological measurements affect sensory perception. For example, compared with imitation tests performed with a texture profile analysis (TPA) device, rheological tests of foods measure the physical properties of the system and do not mimic the human sensory process. Therefore, many simple tests have been developed to measure the physical/textural properties of food substances [83]. Among the studies conducted, the comparison of the use of gelatin with mammalian gelatins, the effect of other added biopolymers on the product, and its compatibility with food products were evaluated [84]. For example, tilapia skin gelatin was used as a replacement for mammalian gelatin in yogurt, and it was determined that fish gelatin had a lower melting and gel formation temperature compared to mammalian gelatin, but it was reported that it had a higher gelatin content. In addition, it was stated that fish and mammalian gelatin showed similar rheological, sensory, and textural properties [85]. In another study, researchers used type A fish gelatin in yogurt as a substitute for mammalian gelatin, combining it with gum arabic, xanthan gum, and k-carrageenan mixtures. It was reported that the fish gelatin-gum arabic mixture increased viscosity and firmness, while the fish gelatin-k-carrageenan mixture increased viscosity [86]. In a similar study, the use of tilapia fish gelatin in low-fat yogurt was evaluated, and it was reported that a xanthan gum-fish gelatin mix can be used in yogurt containing mammalian gelatin [87]. Rahimah et al. used mackerel bone gelatin as a stabilizer in ice cream, and it was stated that the addition of fish gelatin (0.1%) was accepted by the panelist group [88]. In another similar study, gelatin obtained from salmon skin was used as a stabilizer in ice cream and beer production, and it was determined that fish gelatin improved the quality of ice cream and provided better textural properties, while a clearer effect was observed in beer [89]. Choobkar et al. produced functional lozenges using carp skin gelatin, and it was determined that the lozenge containing fish gelatin had a higher protein content than the lozenge containing bovine gelatin; furthermore, the lozenge containing fish gelatin was preferred by consumers [90]. So, gelatin is used in the food industry in confectionery, meat, dairy-based products, beverages, and functional products [91].

Today, 3D food printing is used particularly in the production of personalized food products and in obtaining products with improved nutritional content. Gelatin is ideal for 3D printing due to its compatibility with food. Gelatin-based systems contribute to the production of complex three-dimensional food structures by ensuring structural stability during printing and maintaining shape after printing. This study focuses on research where gelatin is used in 3D food printing. Bulut & Candoğan used 3D printing technology to create a chicken meat-based snack formulation. Hydrocolloid (gelatin) was added to the product formulation, and the effects of gelatin addition and baking on the quality of 3D-printed products were evaluated. According to the results, the optimum conditions for obtaining the best-matched 3D-printed cylindrical product were 110% filament flow rate, 90% feed rate, 0.5 mm nozzle height, and 1.79% gelatin concentration. After optimization, product properties were evaluated in 3D-printed products before and after baking. Adding 1.79% gelatin increased the storage and loss moduli (G′ and G″ values) and complex viscosity, while decreasing the tan δ value (*p* < 0.05), resulting in a more homogeneous and compact microstructure. All analyzed sensory properties of the gelatin-added product received higher scores compared to the control group (*p* < 0.05) [91]. In another study, Wang et al. worked with sturgeon skin gelatin. Gel formation for printing at room temperature is not possible, and due to its low melting and solidification temperatures, it is difficult to maintain its shape at low temperatures after printing, resulting in energy loss. In their study, they achieved gel formation by mixing different carrageenan species (κ-carrageenan: KG; λ-carrageenan: LG; ι-carrageenan: IG) with sturgeon skin gelatin. It was reported that the turbidity of the mixtures increased significantly with the addition of 0.4–0.8% carrageenan. The highest gel strength was determined as 104.02 g when the KG additive concentration was 0.4%, and the hardness and tackiness of the gel increased 5.44 times and 5.37 times, respectively, compared to a single gelatin sample when the KG concentration reached 0.8%. The melting temperature of the mixing system was most significantly improved when IG was added, while LG provided the least improvement. Therefore, carrageenan has a positive effect on improving the gelation properties and application in room-temperature 3D printing of sturgeon skin gelatin systems [92]. Bian et al. conducted a study on the development of composite food hydrogels containing fish gelatin (FG) and high acyl gellan gum (HG) for 3D printing and stated that the composite inks exhibited shear thinning behavior with low viscosity and sufficient mechanical support at high shear speeds. The temperature response of the inks showed their gelation behavior and stability during the printing process. FG-HG composite inks demonstrated suitable print adaptability and the potential to produce complex functional foods with precise structures. During the 3D printing process, the printer temperature was 50 °C while the ambient room temperature was left at 25 °C. With a nozzle diameter of 0.84 mm and a printing speed of 15 mm/s, successful 3D-printed products were created. In addition, the extrusion capability is affected by the viscosity (η) due to the shear stress of the printing ink in the nozzle. In the temperature response of ink formulations with different FG/HG ratios, the ink is subjected not only to shear force but also to significant temperature changes during extrusion from the nozzle. It was determined that the FG-HG composite gel exhibits solid-like behavior. This indicates that increasing the concentration of FG and HG increases the G′, G″, and G* values of the printed ink to varying degrees [38]. Gelatin is considered one of the biomaterials with high potential for use as a support structure for regenerative therapy [93].

In a study by Hu et al. the substance developed by applying an enzymatic/ultrasonic hydrolysis method to obtain chitooligosaccharide was loaded onto gelatin, because gelatin can have a high loading capacity to protect the active ingredients eugenol, tannic acid, and resveratrol, which have various applications, and can function as a fat substitute in mixed yogurt because they have the ability to stabilize yogurt systems and improve the perceived taste in the mouth, giving yogurt a delicate and smooth taste similar to commercial yogurt. Three-dimensional printing was performed to develop special personalization applications, and a yogurt product functionalized with chitooligosaccharide was developed for commercial dairy applications. Composites based on 0.2% weight chitooligosaccharide and 2% weight gelatin were added [94]. In another study, Zhou et al. added peach gum polysaccharide (PGP) to gelatin to prepare curcumin-containing 3D-printed chewing gum confectionery, and rheology tests showed that the addition of PGP could effectively improve apparent viscosity and thermal stability, consequently enhancing the 3D printing and supportability of the products. When the PGP addition was 6%, the printing accuracy was found to be over 90%, and according to the results of texture and microstructure analysis, the addition of PGP promoted the formation of a dense gel structure and increased the water-holding capacity and supportability of the gel materials [95].

## 6. Challenges, Limitations, and Future Perspectives

Gelatin presents several challenges in its use; most fundamentally, because it is of animal origin, it is not suitable for individuals who prefer vegetarian and vegan diets. Another difficulty in using gelatin is its thermal instability, which causes it to melt even at low temperatures, making it unsuitable for applications requiring high heat treatment. Furthermore, the quality characteristics of gelatin vary depending on its production method and source. Finally, there is a growing trend toward alternative animal-based sources [26]. And, in the use of gelatin, considering the variability of raw materials, functional trade-offs, scalability constraints, and challenges related to long-term stability and sustainability, the future development of functionalized gelatin should move beyond experimental trial-and-error approaches toward rational, application-oriented design frameworks [96]. In response to regulatory restrictions and consumer concerns about residual chemicals, research is increasingly shifting toward physical and enzymatic modification pathways, including controlled aggregation, thermal background regulation, non-covalent network reinforcement, and enzyme-mediated cross-linking. These approaches enable functional improvement while maintaining food-grade safety and biocompatibility. Increased utilization of fish and poultry processing by-products can reduce reliance on traditional mammalian sources. At the processing level, integration of green technologies such as ultrasound and electrical or magnetic field-assisted methods into gelatin extraction and modification from these alternative sources offers opportunities to reduce environmental impact while providing additional control over molecular organization and functional performance [96].

The main obstacles in the use of gelatin include temperature sensitivity and gelatin-based drug delivery systems, which are prone to degradation under changing environmental conditions, hindering integrity due to thermo-sensitivity and structural instability, leading to difficulties in drug use and storage. However, the stability of gelatin can be ensured by combining it with thermostable polymers. The most significant disadvantage of gelatin in 3D printing is its temperature sensitivity. This sensitivity causes deformation, reduced print accuracy, and loss of stability, resulting in defective structures and defects in 3D printing. Deformations can occur in microchannel structures, particularly in the biomedical field, when gelatin is used. Storage and stability also affect the use of gelatin because gelatin undergoes degradation under certain conditions and experiences stability issues in encapsulated drug form. Furthermore, variables such as humidity, temperature, and light exposure affect the stability of nanoparticles. Due to its hydrophilic structure, gelatin-containing bioinks are susceptible to microbial degradation, leading to stability issues. This restricts the 3D printing process. Gelatin that hardens during storage causes jamming in the nozzle, while gelatin in gel form at low temperatures causes stability limitations during printing. Another important point is the regulatory concerns of gelatin usage. Concerns regarding the use of gelatin involve risks related to safety, quality control, and the origin and variability of gelatin, as its animal origin and the use of bovine-derived materials carry a risk of transmission of spongiform encephalopathies (e.g., bovine spongiform encephalopathy). Three-dimensional printing, being a new technology, lacks certain standards, especially for gelatin, where the additives used and production parameters must be considered. For example, gelatin-based 3D-printed products used in biomedical fields are classified as medical devices or advanced therapeutic products and require ISO 10993-1:2025 (edition 6, 2025) [97], biological evaluation of medical devices, biocompatibility testing, and sterilization assessments. All these reasons, along with the lack of regulation, are factors that restrict the use of gelatin with 3D printers. Cost-effectiveness is relevant, as gelatin is relatively inexpensive in the food and cosmetic industries; it is costly in advanced biomedical and clinical applications because additional costs are required when material modification and scaling are necessary [17,97]. Also, with vegetarian and vegan lifestyles, the demand for plant-based alternatives has also increased. Therefore, bovine and porcine gelatin is not suitable for those following a plant-based diet. Plant-based alternatives should take this consumer trend into account. Ethical and environmental concerns about animal welfare and environmental impact are important, and there is a shift toward more sustainable and ethical sources and alternative sources that do not rely on animal products, contributing to more ethical and environmentally friendly practices. For this reason, plant-based gelatin alternatives have the advantage of being halal certified and potentially contributing to environmental sustainability by reducing carbon footprints [98]. Another point is that cooking integration is the idea of cooking immediately after printing to eliminate bacteria for food sterilization has been suggested, and even the idea of doing printing and cooking in a single machine has been proposed, using microwaves or infrared heating. However, preserving nutritional value is one of the main challenges, as printing a matrix coated with heat-sensitive antioxidants and then rapidly cooking the printed structure could be considered. In heat-requiring post-processing operations, such as in ovens or microwaves, 3D prints containing gelatin easily lose their shape and geometry. This also occurs when used with hot sauces or similar liquids. Therefore, 3D-printed foods containing gelatin should be kept cold and are suitable for cold consumption. This situation limits the range of gelatin-containing products in 3D printing. This situation restricts the use of gelatin-containing products in 3D printing [56]. Lastly, clean label and consumer acceptance are important obstacles for visibility of ingredients, which is important to consumers because it is important to know the hydrocolloids in the formulations, such as starch, gelatin, kappa carrageenan, and calcium salts. The acceptability of the food is increased by the selection of short, transparent labels that reflect how consumers evaluate the naturalness and acceptability of the ingredients. The phrase “3D-printed food” can be associated with artificiality, industrial processes, and high technology by consumers, making product acceptance more difficult [16].

However, the use of alternatives derived from fish, fruit, and other plant materials allows for the removal of religious restrictions, offering an alternative to mammalian-derived gelatin. The utilization of plant-based alternatives and fish waste contributes to environmental sustainability by potentially reducing carbon footprints and addressing broader ecological concerns. The production of these alternatives generally involves more efficient resource use, lower greenhouse gas emissions, and less environmental impact compared to traditional animal-derived gelatin. However, there are some technical challenges in using gelatin alternatives; most importantly, achieving functional properties like traditional gelatin, such as texture, mouthfeel, and stability, which can be difficult with alternative sources. Furthermore, some plants or marine-derived gelatins may be less common or more expensive than traditional animal-derived gelatin. In addition to sustainability and life cycle assessment research beyond its biomedical potential, fish gelatin also represents a sustainable alternative within the context of the circular economy. Recent life cycle assessment (LCA) studies comparing marine and mammalian gelatin have shown that gelatin derived from fish generally has a lower carbon footprint and less land and water use; this is primarily due to its utilization of waste from fisheries rather than relying on specialized animal husbandry.

The advantages of 3D printing are its ease of use, low cost, efficient production, personalization, suitability for bioprinting, and suitability for functional tissue production. Its disadvantages are the following: consumer acceptance is insufficient; the original form of the printed object is considered, but its dynamic nature is not considered. Through various processing methods, its gel strength and stability are being improved to make it suitable for 3D printing. For example, in the food industry, gelatins with specific gelling capacity, Bloom values, and viscosities are used in products; in the healthcare sector, gelatins are used particularly in bone and dental implants and in packaging. One of the significant advancements in the use of gelatin is the development of more stable alternatives; these include plant-based hydrocolloids such as agar, pectin, gellan gum, and carrageenan, as well as gelatin derived from fish gelatin and its by-products for sustainable and alternative resource utilization. The use of gelatin in the encapsulation and biodegradable packaging industries is also being discussed. Additionally, it may be possible to develop modified gelatin types with high stability and mechanical strength.

The long-term sustainability of the gelatin industry depends on the adoption of novel technologies. As demand for gelatin continues to grow across diverse sectors, innovation is essential to modernize traditional processing methods. Future developments should prioritize economical and environmentally sound technologies while maintaining product quality and functional performance. While these developments continue, improvements related to gelatin’s 3D printing, sterilization, and scalability constraints must be addressed. Therefore, future research should prioritize standardized characterization protocols, green and reproducible synthesis routes, and rigorous in vivo evaluations to ensure safety and regulatory compliance.

## 7. Conclusions

In summary, gelatins derived from fish and edible insects have provided an acceptable alternative source for halal and kosher products and offered an alternative solution for markets concerned about bovine spongiform encephalopathy (BSE). Furthermore, fish gelatin has lower gel strength, a lower melting point, and lower proline and hydroxyproline content compared to bovine and porcine gelatin. The rheological properties of fish gelatin, including gel strength, viscosity, and other rheological characteristics, differ from those of mammalian gelatins. Particularly due to its low cost, wide availability, biocompatibility, degradability, gel-forming capacity, and food compatibility, the use of fish gelatin has been shown in recent years as a very powerful production tool in 3D printing, biomedical, food, and healthcare fields. Production occurs through layer-by-layer deposition, aiming for efficient use of proteins and living cells. This type of production allows for the creation of personalized products at low cost. One of its most important advantages is the ability to produce devices and nutrients tailored to the individual needs of patients, thus providing them with a more comfortable life. Commonly used 3D printing technologies in biomedical applications include fused deposition modeling (FDM), stereolithography (SLA), selective laser sintering (SLS), electron beam melting (EBM), and direct metal laser sintering (DMLS). In food production, 3D printing technology is mostly extrusion-based (Fusion Additive Modeling and Direct Ink Printing), SLS, SLA, and inkjet printing. Given its widespread use, the inclusion of gelatin in 3D printing processes is valuable. Among the various valuable products obtained from waste, gelatin extraction is an ideal way to utilize waste due to its effective applications in the biomedical and pharmaceutical industries. The relationship between 3D printing and gelatin is primarily influenced by rheological properties such as gel strength and viscosity, as well as thermal stability and solubility, such as melting and gelling degree, and fundamental physicochemical properties such as color, transparency, taste, and odor, which determine the quality of the gelatin. Another important issue in bioink and 3D bioprinting processes is sterilization to prevent contamination, ensure cell viability, product safety, and the accuracy of experimental results. The sterilization methods used include hot air oven, autoclave, pasteurization, electron beam, ultraviolet rays, syringe filtration, plasma, ethylene oxide, ozone, formaldehyde, hydrogen peroxide, sodium hypochlorite, peracetic acid, ethanol, and membrane filtration.

Gelatin-based 3D printing technologies represent a highly versatile and promising platform across biomedical engineering, food science, and health-related applications. Gelatin’s unique combination of biocompatibility, gelling ability, tunable textural properties, and wide availability makes it particularly suitable for extrusion-based additive manufacturing. Current research demonstrates its successful integration into diverse systems, including tissue-engineering scaffolds, controlled drug-delivery matrices, and customized food products.

Despite these advantages, challenges remain. Gelatin’s animal-derived origin, batch-to-batch variability, and limited thermal stability can restrict its performance in highly controlled printing environments. Because extrusion behavior is strongly governed by rheological parameters such as viscosity, shear-thinning response, viscoelasticity, and gelation dynamics, optimizing these properties is essential to achieving reproducible and high-resolution printed structures.

The long-term sustainability of the gelatin industry depends on the adoption of new technologies. As demand for gelatin continues to grow across various sectors, innovation is essential to modernize traditional processing methods. Future developments should prioritize economical and environmentally friendly technologies while maintaining product quality and functional performance. As these developments continue, improvements related to the sterilization and scalability constraints of 3D printing gelatin must be addressed. Therefore, future research should prioritize standardized characterization protocols, green and reproducible synthesis pathways, and rigorous in vivo evaluations to ensure safety and regulatory compliance.

However, our literature review revealed that studies on gels and 3D printing in various food, health, and biomedical fields have investigated different formulations and 3D printing technologies. These studies focused on rheological properties such as printability and mechanical strength, examining the conditions and formulations that yield successful results. However, a lack of systematic comparison between these studies has resulted in the existing literature remaining descriptive and failing to reach a critical evaluation level. Properties such as temperature, strength, printability, shape stability, and stability have been listed, but their inclusion in the 3D printing process has not been discussed in detail. Problems such as gelling behavior and nozzle flow disturbances during pressing are present in the relationship between gelatin and temperature. These issues do not necessarily require a more detailed examination of the relationship between the printing process parameters.

## Figures and Tables

**Table 1:** Physical properties and advantages of different types of gelatin [15,26].

Type of Gelatin	Gel Strength	Melting Point	Viscosity	Advantages	Disadvantages	Applications
Fish Gelatin	50–150 g	25–30 °C	1.5–4.0	Sustainable Cultural acceptance	Low gel strength Quality variable of source and process	Drug delivery Scaffold Food
Bovine Gelatin	200–300 g	35–40 °C	2.0–7.0	High gel strength	Ethical and religious problems Zoonotic risk	Drug delivery Food
Porc Gelatin	200–300 g	35–40 °C	2.0–6.0	High gel strength	Ethical and religious problems Zoonotic risk	Drug delivery Food
Plant based alternatives	Depends on polymer	Depends on polymer	Depends on polymer	Abundant No ethical problem	Modification procedures depend on the polymer	Food packaging Biomedical usage

**Table 2:** Advantages and disadvantages of gelatin [6,14,15,26,27,28].

Advantages of Gelatin	Disadvantages of Gelatin
Excellent gelling availability and stabilization properties in food	Dietary limitations due to animal origin
Chewable, elastic texture and high sensory effect	Religious restrictions
Thermoreversible—melt structure	Sensitive to temperature, pH and enzymes
Wide range of applications and low cost	Long setting time
High biocompatibility	Need support for stability and mechanical strength

**Table 3:** Gelatin-based applications of 3D printing.

Technology Parameters	Bioink Formulation	Printing Objective/Structure	Application	Functional Properties	Ref.
One-nozzle extrusion-based 3D bioprinting	Gelatin and hepatocyte mix	3D structure	Artificial human tissues and organs	Support viable and performed biological functions	[32]
One-nozzle extrusion-based 3D bioprinting	Gelatin and chitosan and hepatocyte mix	3D architectural design	Organ manufacturing	Support living cells	[33]
One-nozzle extrusion-based 3D bioprinting	Gelatin and hyaluronan mix	3D hydrogel structures	Brain defect repair	Support biocompatibility for brain tissue	[34]
One-nozzle extrusion-based 3D bioprinting	Gelatin/alginate and myoblast mix	-	Muscles	Help cell growth	[35]
3D bioprinting	Fish scale gelatin and alginate	3D cell scaffold	Tissue engineering and wound dressing	Biocompatibility and non-toxicity	[36]
3D bioprinter	Gelatin and alginate mix	Grid-like structures	Biomedical applications regeneration and repair bones	Shape fidelity and stability	[37]
Extrusion-based 3D printing	Fish gelatin and high acyl gellan gum mix	butterfly, turtle, flower, mouse and cat structures	Food application	Strong potential for production of functional food	[38]
Microwave 3D printing	Surimi paste with gelatin and κ-carrageenan mix	Solid cylinders and hollow spheres	Food application	Improved surimi gel structure	[39]

## Data Availability

No new data were created or analyzed in this study.

## Supplementary Materials

The following supporting information can be downloaded at: https://www.mdpi.com/article/10.3390/md24060217/s1. Figure S1: Examples of gelatin-based 3D printing applications in food industries; (A) microalgae residue gelatin Pickering gels. Figure S2: Examples of gelatin-based 3D printing applications in biomedical industries.

## Abbreviations

The following abbreviations are used in this manuscript:

2D	Two-dimensional
3D	Three-dimensional
FG	Fish gelatin
SGG	Salmon skin gelatin
ADSCs	Adipose-derived stem cells
CS-Gel	Chitosan–gelatin
GelMA	Gelatin methacrylate
PGP	Peach gum polysaccharide
EBM	Electron beam melting
MTT assay	3-(4,5-dimethylthiazol-2-yl)-2,5-diphenyl tetrazolium bromide assay
mTOR	Mechanistic target of rapamycin
SLS	Selective laser sintering
STL	Stereolithography
FDMSLS	Fused layered modeling
DMLS	Direct metal laser sintering
SLA	Stereolithography
