# Supplementary material for: 3D Printing with Marine Gelatin: A Cross-Sector Review of Biomedical, Food, and Health Uses
Source: Mar Drugs. 2026 Jun 16;24(6):217. doi: 10.3390/md24060217 (PMC13301568; doi:10.3390/md24060217)
Supplement: Supplementary file 1 [file marinedrugs-24-00217-s001.zip › marinedrugs-4335917-supplementary.pdf]

## Supplementary File

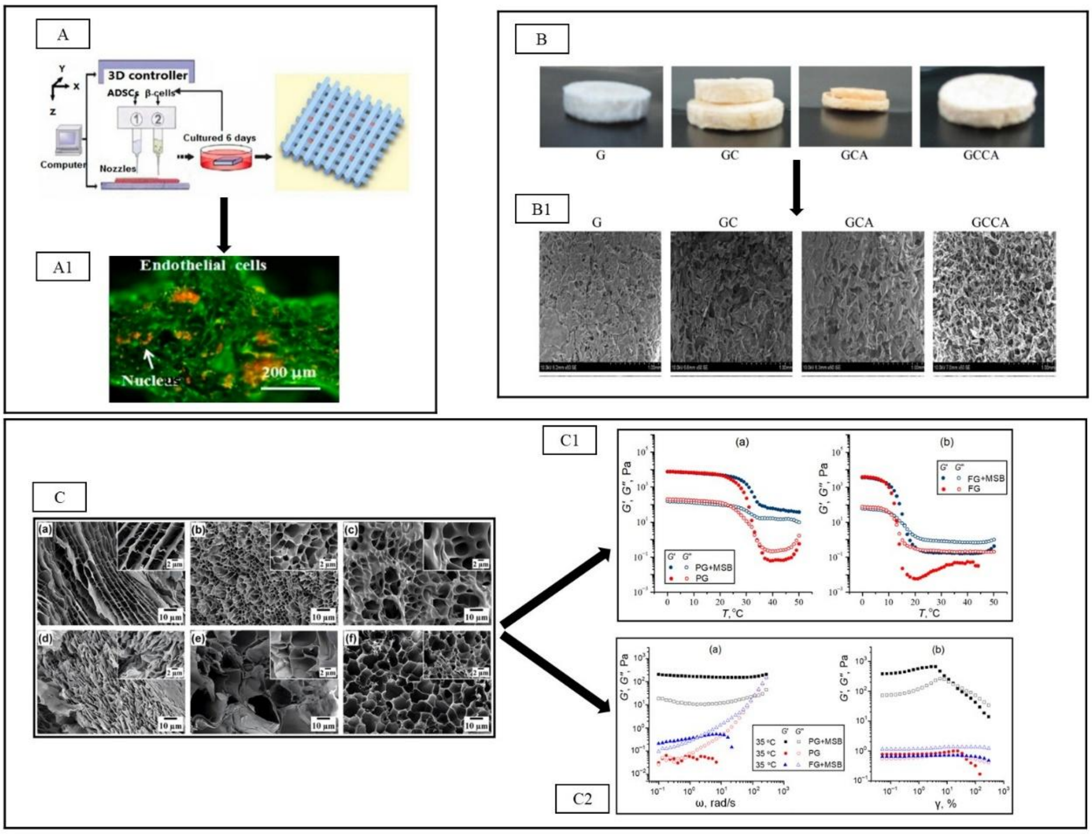


Figure S1. Examples of gelatin-based 3D printing applications in biomedical industries. (A) Gelatin-based hydrogel organ created by 3D bioprinting technology - Construction process of a gelatin/alginate/fibrin hydrogel mix loaded with fat-derived stem cells and vascularized organ of β-cells under 3D bioprinting [71]; (A1) Immunostaining image of 3D printed structure with mAbs in green for CD31+ cells and pyrindin in red for cell nuclei[98]; (B) Images of different types of fish skin gelatin scaffolds (G: simple gelatin; GC: gelatin + chitosan; GCA: gelatin + calcium acetate; GCCA: gelatin + chitosan + calcium acetate) [19]; B1: SEM microstructure of gelatin mixes (G: simple gelatin; GC: gelatin + chitosan; GCA: gelatin + calcium acetate; GCCA: gelatin + chitosan + calcium acetate) [19]; (C) SEM images of fish gelatin and mammalian gelatin: FG (a), FG (10%) after freeze-thaw cycle (b), FG (10%) cross-linked with MSB (c), PG (2%) (d), 10% PG (10%) after freeze-thaw cycle (e), 10% PG (10%) cross-linked with MSB (f) [72]; (C1) Temperature sweeps of mammalian (a) and fish (b) gelatin hydrogels: filled symbols represent G′; empty symbols represent G″ [72]; (C2) Dependence of G′ - storage modulus - and G″ - loss modulus - of mammalian and fish gelatin hydrogels and of uncross-linked mammalian gelatin hydrogel (35 °C) on frequency ω (a) at γ = 1% and dependence on strain γ at f = 1 Hz (b); filled symbols represent G′; empty symbols represent G″[99].

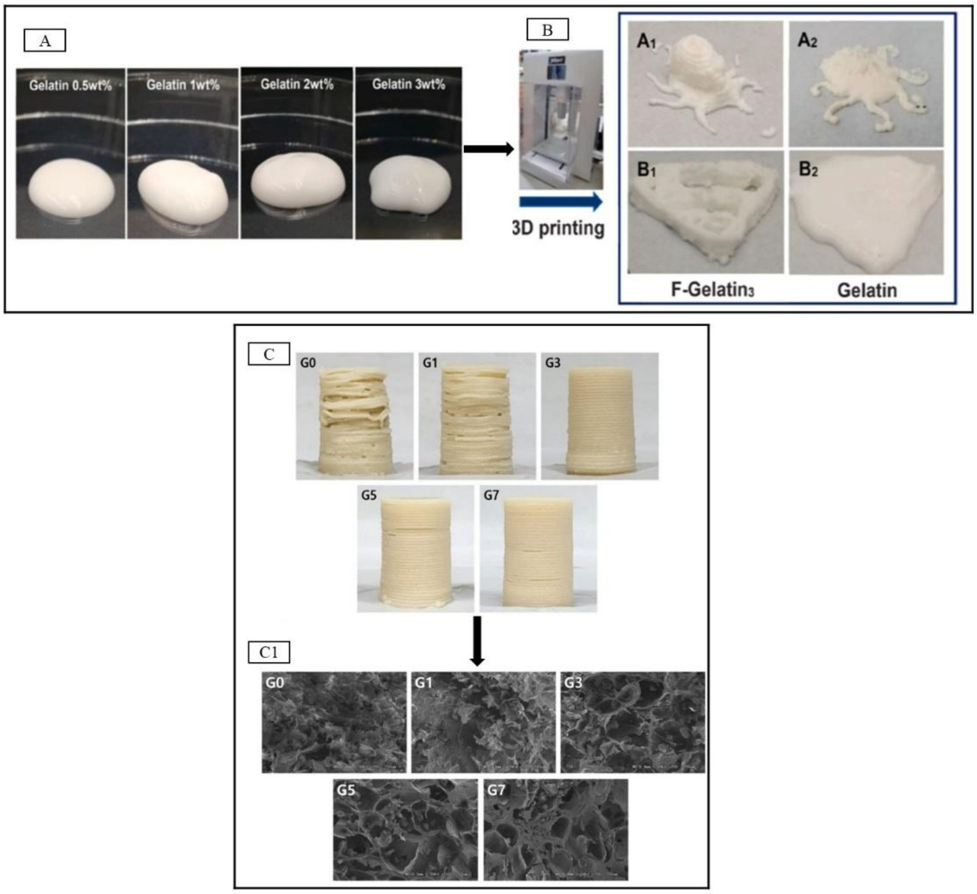


Figure S2. Examples of gelatin-based 3D printing applications in food industries (A) Microalgae residue gelatin Pickering gels [16]; (B) 3D printing of microalgae residue-gelatin Pickering gels [16]. (C); Cylindrical printing tests of 3D printing ink for gelatin at different concentrations: G0 - 0%; G1 - 1%; G3 - 3%; G5 - 5%; G7 - 7% [80]; (C1) Scanning electron microscope (SEM) images of 3D printing ink for gelatin (G0, 0%; G1, 1%; G3, 3%; G5, 5%; G7, 7%) [80].
